# Correction: A theory of discrete hierarchies as optimal cost-adjusted productivity organisations

**DOI:** 10.1371/journal.pone.0218010

**Published:** 2019-05-31

**Authors:** Sandro Claudio Lera, Didier Sornette

Fig 1 was converted incorrectly. The authors have provided a corrected version here.

**Fig 1:**
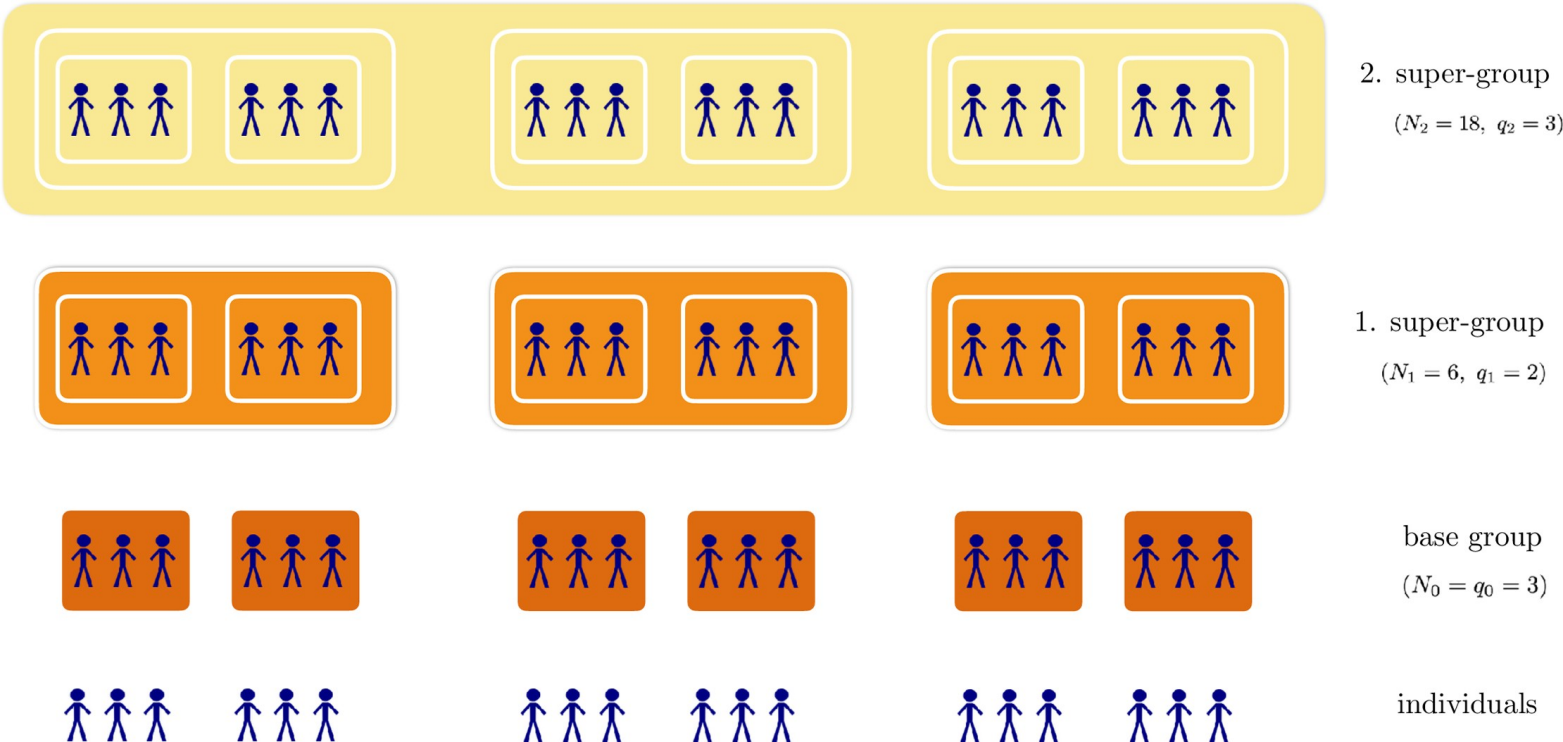
Illustration of the hierarchical organization. We show the case with *p* = 2, i.e. 3 hierarchical levels (without counting the individual level). We start from *N* = 18 individuals. The base groups are of size 3 (*N*_0_ = *q*_0_ = 3), i.e. three individuals together form one group. The next groups (the first super-groups) are of size 2 (*q*_1_ = 2, *N*_1_ = *q*_1_ ⋅ *q*_0_ = 6), i.e. two base-groups together form one higher order group. The second super-group (and also the top level) is again of size 3 (*q*_2_ = 3, *N*_2_ = *q*_0_ ⋅ *q*_1_ ⋅ *q*_2_ = *N* = 18).
