# Supplementary material for: A theory of discrete hierarchies as optimal cost-adjusted productivity organisations
Source: PLoS One. 2019 Apr 18;14(4):e0214911. doi: 10.1371/journal.pone.0214911 (PMC6472750; doi:10.1371/journal.pone.0214911)
Supplement: S1 Fig — (PDF) [file pone.0214911.s001.pdf]

A Theory of Discrete Hierarchies as Optimal Cost-Adjusted Productivity Organisations: Supplementary Information

Picking up the notations from the main paper, we show here various additional results that have been mentioned there.

1 Recursive optimal group sizes

In this section, we optimise the output of the hierarchically structure

$$\Pi = \sum_{r=0}^{p} \Big(\mu_r q_r^{\beta} - \lambda_r q_r (q_r - 1) \Big) \times \frac{N}{\prod_{i=0}^{r} q_i} \tag{1}$$

analytically for a fixed number of hierarchical levels $p \in \{0, \ldots, p_{\max}\}$. To this end, we first rewrite (1) in terms of the number of people in the r-th super-group N_r, where $q_r = N_r/N_{r-1}$, which gives

$$\frac{\Pi}{N} = \sum_{r=0}^{p} \left(\mu_r \frac{N_r^{\beta-1}}{N_{r-1}^{\beta}} - \lambda_r \frac{N_r}{N_{r-1}^2} + \lambda_r \frac{1}{N_{r-1}} \right). \tag{2}$$

We now find the optimum by setting the derivative of (2) with respect to N_r to zero, i.e.

$$\begin{aligned} 0 &= \frac{\partial}{\partial N_r} \left(\ldots + \mu_r \frac{N_r^{\beta-1}}{N_{r-1}^{\beta}} + \mu_{r+1} \frac{N_{r+1}^{\beta-1}}{N_r^{\beta}} + \ldots - \lambda_r \frac{N_r}{N_{r-1}^2} - \lambda_{r+1} \frac{N_{r+1}}{N_r^2} + \ldots + \lambda_{r+1} \frac{1}{N_r} \right) \\ &= \mu_r (\beta - 1) \frac{N_r^{\beta-2}}{N_{r-1}^{\beta}} - \mu_{r+1} \beta \frac{N_{r+1}^{\beta-1}}{N_r^{\beta+1}} - \lambda_r \frac{1}{N_{r-1}^2} - 2\lambda_{r+1} \frac{N_{r+1}}{N_r^3} - \lambda_{r+1} \frac{1}{N_r^2}. \end{aligned}$$

Multiplying by N_r and restoring the notation $q_r = N_r/N_{r-1}$, we then find

$$0 = \mu_r (\beta - 1) q_r^{\beta} - \mu_{r+1} \beta q_{r+1}^{\beta-1} - \lambda_r q_r^2 - 2\lambda_{r+1} q_{r+1} - \lambda_{r+1}, \tag{3}$$

which, after rearranging terms, yields the recursive equation

$$\mu_{r+1} \beta q_{r+1}^{\beta-1} + 2\lambda_{r+1} q_{r+1} = \mu_r (\beta - 1) q_r^{\beta} - \lambda_r q_r^2 - \lambda_{r+1}. \tag{4}$$

For general β, this iterative equation has to be solved numerically. Additionally, the constraints

$$q_r \in (2, N/2^p) \qquad \text{and} \qquad N = \prod_{r=0}^{p} q_r \tag{5}$$

apply. Hence, it is easier to just optimise (1) numerically, as described in the next section. A special case ("military hierarchy") is treated analytically in section 3.

2 Numerical optimisation of the production

In this section, we describe how we have optimised expression (1) numerically. For fixed $p \in \{0, \dots, p_{\max}\}$, we are looking for the optimal vector of group sizes $(q_0, \dots, q_p)$, subject to the constraints (5). To this end, we have used a Sequential Least Squares Programming (SLSQP) function, as first described in [2] and implemented in Python's SciPy [1], as it can handle both constraints and variable bounds. To avoid being stuck in local minima, we first sample some 100 initial guesses, subject to constraints (5). We then call SLSQP for each of these 100 initial values in parallel, and finally select the global maximum.

We repeat this procedure for every $p \in \{0, \dots, p_{\max}\}$. Through direct comparison, we finally find the optimal $p^* = p$ that maximises expression (1).

3 Analytical treatment of the "military hierarchy"

In this section, we treat the case of a "military hierarchy" analytically, i.e. we consider the configurations that maximise production (1) with $\mu_r = \omega\delta_{r0}, \lambda_r = \rho^r$. The fact that $\mu_{r>0} = 0$ should not be construed as meaning that the higher levels of the hierarchy are useless because they do not contribute directly to production. As we shall see, the higher levels are needed to minimise coordination costs as in Ref.[3], so that the higher levels of the organisation can be interpreted as helping optimise resource allocation and management to empower the bottom level in its production tasks.

In contrast to general geometrical hierarchies, the military hierarchy allows for an analytical treatment, as long as we are working with the approximation $q_r(q_r - 1) \approx q_r^2$, which is valid for q_r not too small. For fixed p, and recalling that $q_r = N_r/N_{r-1}$, we find the value $N_r = \prod_{i=0}^r q_i$ that maximises

$$\Pi = \sum_{r=0}^{p} \left(\mu_r q_r^\beta - \lambda_r q_r^2\right) \times \frac{N}{\prod_{i=0}^r q_i} \tag{6}$$

as

$$0 \stackrel{!}{=} \frac{\partial \Pi}{\partial N_r} \tag{7a}$$

$$= N \frac{\partial}{\partial N_r} \left[\omega N_0^{\beta-1} - \sum_{r=0}^{p} \lambda_r \frac{N_r}{N_{r-1}^2}\right] \tag{7b}$$

$$\Updownarrow$$

$$0 = \frac{\partial}{\partial N_r} \left[\omega N_0^{\beta-1} - \sum_{r=0}^{p} \lambda_r \frac{N_r}{N_{r-1}^2}\right] \tag{7c}$$

$$= \omega(\beta-1)N_0^{\beta-2}\delta_{r0} - \frac{\partial}{\partial N_r}\left(\dots + \lambda_r \frac{N_r}{N_{r-1}^2} + \lambda_{r+1}\frac{N_{r+1}}{N_r^2} + \dots\right) \tag{7d}$$

$$= \omega(\beta-1)N_0^{\beta-2}\delta_{r0} - \lambda_r \frac{1}{N_{r-1}^2} + 2\lambda_{r+1}\frac{N_{r+1}}{N_r^3} \tag{7e}$$

$$\Updownarrow \quad \text{(multiply by } N_r^2\text{)}$$

$$0 = \omega(\beta-1)N_0^{\beta}\delta_{r0} - \lambda_r \frac{N_r^2}{N_{r-1}^2} + 2\lambda_{r+1}\frac{N_{r+1}}{N_r} \tag{7f}$$

$$= \omega(\beta-1)q_0^{\beta}\delta_{r0} - \lambda_r q_r^2 + 2\lambda_{r+1}q_{r+1} \tag{7g}$$

$$\Updownarrow \quad \text{(divide by } 2\lambda_{r+1}\text{)}$$

$$0 = \frac{1}{2}(\beta-1)\frac{\omega}{\lambda_1}q_0^{\beta}\delta_{r0} - \frac{1}{2}\frac{\lambda_r}{\lambda_{r+1}}q_r^2 + q_{r+1}, \tag{7h}$$

resulting in the recursive relation

$$q_{r+1} = \frac{1}{2\rho} q_r^2 - \frac{1}{2}(\beta - 1)\frac{\omega}{\rho} q_0^\beta \delta_{0r}. \tag{8}$$

Plugging (8) into (6), we obtain the following expression for the overall production:

$$\begin{aligned}
\Pi(p)/N &= \sum_{r=0}^{p} \left(\mu_r q_r^\beta - \lambda_r q_r^2\right) \times \frac{1}{N_r} && \text{(9a)}\\
&= \sum_{r=0}^{p} \left(\mu_r q_r^\beta - \lambda_r q_r^2\right) \times \frac{1}{\prod_{i=0}^{r} q_i} && \text{(9b)}\\
&= \omega q_0^{\beta-1} - \sum_{r=0}^{p} \rho^r \frac{q_r^2}{q_0 \cdot q_1 \cdot \ldots \cdot q_r} && \text{(9c)}\\
&= \omega q_0^{\beta-1} - q_0 - \sum_{r=1}^{p} \rho^r \frac{q_r^2}{q_0 \cdot q_1 \cdot \ldots \cdot q_r} && \text{(9d)}\\
&= \omega q_0^{\beta-1} - q_0 - \sum_{r=1}^{p} \rho^r \frac{q_r}{q_0 \cdot q_1 \cdot \ldots \cdot q_{r-1}} && \text{(9e)}\\
&= \omega q_0^{\beta-1} - q_0 - \sum_{r=1}^{p} \rho^r \frac{1}{2\rho} \frac{q_{r-1}^2}{q_0 \cdot q_1 \cdot \ldots \cdot q_{r-1}} && \text{(9f)}\\
&= \omega q_0^{\beta-1} - q_0 - \sum_{r=1}^{p} \rho^r \frac{1}{2\rho} \frac{q_{r-1}}{q_0 \cdot q_1 \cdot \ldots \cdot q_{r-2}} && \text{(9g)}\\
&\vdots\\
&= \omega q_0^{\beta-1} - q_0 - \sum_{r=1}^{p} \rho^r \frac{1}{(2\rho)^{r-1}} \frac{q_1}{q_0} && \text{(9h)}\\
&= \omega q_0^{\beta-1} - q_0 - \sum_{r=1}^{p} \rho^r \frac{1}{(2\rho)^{r-1}} \left[\frac{1}{2\rho} q_0 - \frac{1}{2}(\beta-1)\frac{\omega}{\rho} q_0^{\beta-1}\right] && \text{(9i)}\\
&= \omega q_0^{\beta-1} - q_0 - \sum_{r=1}^{p} \rho^r \frac{1}{(2\rho)^r} \left[q_0 - (\beta-1)\ \omega\ q_0^{\beta-1}\right] && \text{(9j)}\\
&= \omega q_0^{\beta-1} - q_0 - \sum_{r=1}^{p} \frac{1}{2^r} \left[q_0 - (\beta-1)\ \omega\ q_0^{\beta-1}\right] && \text{(9k)}\\
&= \omega q_0^{\beta-1} - q_0 - \left[1 - \left(\frac{1}{2}\right)^p\right] \left[q_0 - (\beta-1)\ \omega\ q_0^{\beta-1}\right]. && \text{(9l)}
\end{aligned}$$

Since $N_0 = q_0$, the maximal production for fixed p is thus given by

$$\Pi(p) = \omega N N_0^{\beta-1} - N_0 N - N \left[1 - \left(\frac{1}{2}\right)^p\right] \left[N_0 - (\beta-1)\omega N_0^{\beta-1}\right]. \tag{10}$$

We check that for $p = 0$ we recover $\omega N^\beta - N^2$. The size of the base group $N_0 = q_0$ is not a

free parameter, but fixed by N. To calculate the relation between N_0 and N, note that

$$
\begin{aligned}
q_r &= \frac{1}{2\rho} q_{r-1}^2 - \frac{1}{2}(\beta-1)\frac{\omega}{\rho} q_0^\beta \delta_{r1} && \text{(11a)}\\
&\vdots \\
&= \frac{1}{(2\rho)^{\sum_{k=0}^{r-2} 2^k}} q_1^{2^{r-1}} && \text{(11b)}\\
&= \frac{1}{(2\rho)^{2^{r-1}-1}} q_1^{2^{r-1}} && \text{(11c)}\\
&= \frac{1}{(2\rho)^{2^{r-1}-1}} \left(\frac{1}{2\rho} N_0^2 - \frac{1}{2}(\beta-1)\frac{\omega}{\rho} N_0^\beta\right)^{2^{r-1}} && \text{(11d)}\\
&= \frac{1}{(2\rho)^{2^r-1}} \left(N_0^2 - (\beta-1)\omega N_0^\beta\right)^{2^{r-1}} && \text{(11e)}
\end{aligned}
$$

for $r \geqslant 1$. Hence,

$$
\begin{aligned}
N &= \prod_{i=0}^{p} q_i && \text{(12a)}\\
&= q_0 \prod_{i=1}^{p} q_i && \text{(12b)}\\
&= N_0 \prod_{r=1}^{p} \frac{1}{(2\rho)^{2^r-1}} \left(N_0^2 - (\beta-1)\ \omega\ N_0^\beta\right)^{2^{r-1}} && \text{(12c)}\\
&= N_0 \frac{1}{(2\rho)^{\sum_{r=1}^{p} 2^r-1}} \left(N_0^2 - (\beta-1)\ \omega\ N_0^\beta\right)^{\sum_{r=1}^{p} 2^{r-1}} && \text{(12d)}\\
&= \frac{1}{(2\rho)^{2^{p+1}-p-2}} N_0 \left(N_0^2 - (\beta-1)\ \omega\ N_0^\beta\right)^{2^p-1}. && \text{(12e)}
\end{aligned}
$$

This equation can be solved numerically to determine N_0 as a function of N, ρ, ω and β. To avoid encountering huge values in the numerical solver, it is useful to rewrite (12e) as

$$(2\rho)^{\frac{2^{p+1}-p-2}{2^p-1}} N^{\frac{1}{2^p-1}} = N_0^{\frac{1}{2^p-1}} \left(N_0^2 - (\beta-1)\ \omega\ N_0^\beta\right). \tag{13}$$

A special case for which (13) can be solved analytically is $\beta = 1$, in which case

$$N_0 = (2\rho)^{\frac{2^{p+1}-p-2}{2^p-1}} \cdot N^{\frac{1}{2^{p+1}-1}}. \tag{14}$$

We also note that, when $\beta = 1$, the recursive relation (8) reduces to the optimal configuration that minimizes the communication overhead. In this case, for $N = 4096$, $N_0 = 16$ for $p = 1$, $N_0 = 3.28$ for $p = 2$, $N_0 = 1.74$ for $p = 3$ and $N_0 = 1.27$ for $p = 4$.

We shall see in the next section that, when cost is minimized, the optimal number of hierarchies p^* scales like $\log(N)$. Plugging $p^* \sim \log(N)$ into (14) we see that N_0 is asymptotically independent of N, since $\lim_{N\to\infty} N^{1/N} = 1$. Thus, in the limit where N_0 is independent of N, and $1 \gg 2^{-p}$, the production (10) simplifies to

$$\Pi(N) = \left(\beta\omega N_0^{\beta-1} - 2N_0\right) \cdot N. \tag{15}$$

For the case $\beta = 1.5, \rho = 0.5, \omega = 6, N = 4096$, the optimal production is $\Pi = 40'447$ with $p^* = 4$ and the optimal structure is given by $(q_0 \approx 9.1, q_1 \approx 2.8, q_2 \approx 2.8, q_3 \approx 6.5, q_4 \approx 8.9)$.

These results are obtained from the numerical optimization using the exact number $q_r(q_r - 1)$ (instead of q_r^2) of interactions and thus are slightly different from the analytical formula, because the approximation $q_r^2 \sim q_r(q_r - 1)$ does not hold very well for the smallest group sizes. Note also that these numbers ($q_0 \approx 9.1, q_1 \approx 2.8, q_2 \approx 2.8, q_3 \approx 6.5, q_4 \approx 8.9$) do not multiply exactly to $N = 4096$ due to rounding errors as we use only one digit after the comma.

Figure 1 shows the total production $\Pi(p)$ as a function of the number p of hierarchical levels minus one for four different values of the production parameter ω for a total population of $2^{12} = 4096$ collaborators. The case $\omega = 6$ is represented in green and is qualitatively similar to the solution obtained for a larger production parameter $\omega = 10$: in both cases, larger subgroups are favoured, especially at the bottom and top levels, with relatively fewer levels ($p^* = 4$ and 3 respectively). In contrast, for smaller production parameters $\omega = 0.5$ and 2, whose legends are shown on the left of the figure, the optimal structure consists in having maximally fragmented hierarchical structures with a maximum number of levels and smallest group sizes (all q_r's are equal to the minimum size 2). The qualitative differences between the hierarchical structures for large versus low production parameter ω illustrate the fight between having large groups at the bottom to enhance productivity and the cost of coordination.

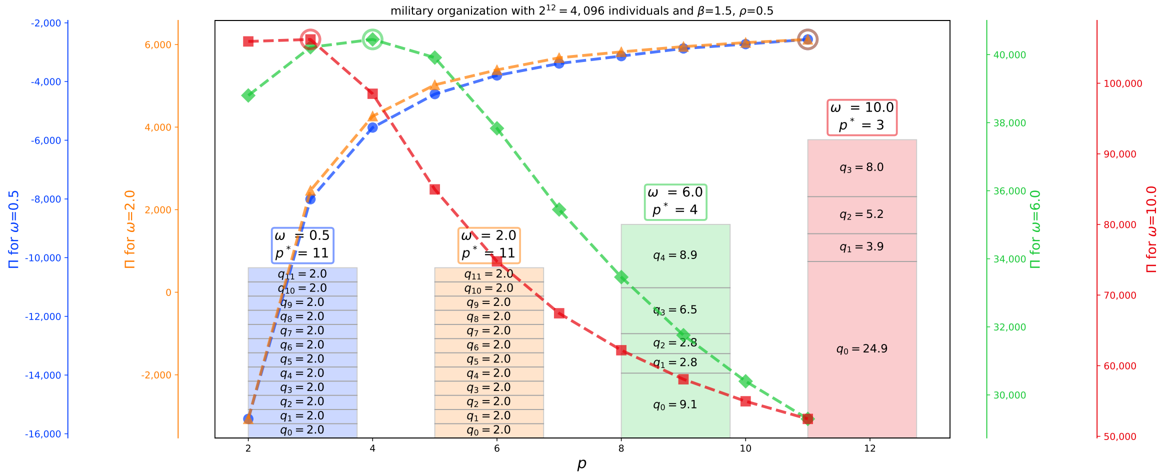

Figure 1: Same as figure 2 in the main text for the military hierarchy defined by $\mu_r = \omega\delta_{r0}$ and $\lambda_r = \rho^r$.

Production $\Pi(p)$ as a function of p (number of hierarchical levels minus one) for four different sets of parameters for a total population of $2^{12} = 4096$ collaborators. For each of the four parameter set indicated in the four legends along the vertical axis, we obtain the optimal group sizes $\{q_0, \ldots, q_p\}$, and calculate the corresponding total production $\Pi(p)$ from expression (6) in the main text. The function $\Pi(p)$ exhibits a maximum at some $p = p^*$ indicated with an open circle. For each of the four optima, the corresponding optimal group sizes $\{q_0, \ldots, q_{p^*}\}$ are given in the form of a stack of rectangles put on top of each other. The four different sets of parameters span different regimes and thus hierarchical designs. Non-integer values of q_r's should be interpreted as a combination of group of integer numbers of collaborators, with numbers within one unit from the quoted q_r and such their average value is as close as possible to the q_r. For instance, $q_r = 3.7$ or 3.8 should be interpreted as corresponding to three groups of 4 and one group of 3.

4 Scaling of the communication overhead

In this section, we consider the asymptotic scaling of the communication overhead as a function of the number of individuals. So instead of maximising production, defined as output minus cost, here we just minimise the cost. This can be seen as a continuation of the work of Toulouse and Bok [3], who calculate the optimal configuration that minimises cost of communication as a function of the base-group size N_0, and for fixed number of hierarchies p. Instead, we find it more natural to consider N as given, and then to ask what is the optimal hierarchical configuration that maximises production. We calculate the communication overhead Γ over $p+1$ hierarchical levels as

$$\begin{aligned}\Gamma(p) &= \sum_{r=0}^{p} (\text{cost between } q_r \text{ subgroups}) \times (\text{number of such groups}) \\ &= \sum_{r=0}^{p} \lambda_r q_r^2 \times \frac{N}{N_r}. \end{aligned} \tag{16}$$

For fixed p (with $p \geqslant 1$), the communication difficulty Γ can be minimized with respect to the group size N_r. Repeating the calculation (7) from section 3 but omitting the output term, we find

$$q_r = \frac{q_{r-1}^2}{2\rho} \qquad \text{for } r = 1, \ldots, p. \tag{17}$$

Using that $N_r = \prod_{i=0}^{r} q_r$, the communication difficulty is calculated by plugging (17) into (16), giving (cf. also (9) for intermediate steps)

$$\Gamma(p) = N \sum_{r=0}^{p} \rho^r \frac{1}{2\rho} \frac{q_{r-1}}{q_{r-2} \ldots q_0} = \ldots = N N_0 \sum_{r=0}^{p} \rho^r \frac{1}{(2\rho)^r} = N N_0 \left(2 - 2^{-p}\right). \tag{18}$$

Similarly to the calculations (11) and (12), the total number of individuals reads

$$N = \prod_{r=0}^{p} q_r = \prod_{r=0}^{p} (2\rho)^{-\sum_{i=0}^{r-1} 2^i} N_0^{2^r} = \prod_{r=0}^{p} (2\rho)^{1-2^r} N_0^{2^r} = (2\rho)^{2+p-2^{p+1}} N_0^{2^{p+1}-1}, \tag{19}$$

which we can invert to find

$$q_0 = N_0 = (2\rho)^{\frac{2^{p+1}-p-2}{2^{p+1}-1}} N^{\frac{1}{2^{p+1}-1}}. \tag{20}$$

Plugging (20) into (18) finally yields

$$\Gamma(p) = \left(2 - 2^{-p}\right) (2\rho)^{\frac{2^{p+1}-p-2}{2^{p+1}-1}} N^{\frac{1}{1-2^{-(p+1)}}}. \tag{21}$$

In particular, for $p = 0$ we recover $\Gamma(0) = N^2$, as anticipated, and as long as $2^{p+1} \gg p$, we may approximate (21) by

$$\Gamma(p) \sim 4\rho N, \tag{22}$$

meaning that the initially quadratically scaling communication difficulty is asymptotically reduced to a linear scaling relation, as long as there are enough hierarchical levels (cf. also the example in section 2 of the main paper, where already for $p = 1$ we find $\Gamma(1) \sim N^{4/3}$).

We aim at minimising (21) with respect to p. Instead of taking the derivative with respect to p, we first write (21) as

$$\Gamma(p) = 2\left(1 - \frac{1}{2}\frac{1}{2^p}\right) (2\rho)^{\frac{2 \cdot 2^p - \log_2(2^p) - 2}{2 \cdot 2^p - 1}} N^{\frac{1}{1-\frac{1}{2}\frac{1}{2^p}}} \tag{23}$$

and introduce the variable $x \equiv 2^p$, leading to

$$\log_2(\Gamma) = \frac{1}{1-\frac{1}{2x}}\log_2 N + \frac{2x - \log_2(x) - 2}{2x-1}\log_2(2\rho) + \log_2\left(1 - \frac{1}{2x}\right) + 1. \tag{24}$$

Introducing furthermore $y \equiv 1 - 1/(2x)$ gives

$$\log_2(\Gamma) = \frac{\log_2 N}{y} + \frac{1 - (1-y)\log_2\left(\frac{1}{2}\frac{1}{1-y}\right) - 2(1-y)}{y}\log_2(2\rho) + \log_2(y) + 1. \tag{25}$$

Because $y = 1 - 1/2^{p+1}$, y varies from $1/2$ to 1 for p varying from 0 to infinity. Already for $p = 2$ we have $y(p = 2) = 0.875$ which is only 12% below 1. Thus, the range of interest is $y = 1 - \epsilon$, where $\epsilon < 0.1$, typically. Replacing y by $1 - \epsilon$ in (25) yields

$$\log_2(\Gamma) = \frac{\log_2 N}{1-\epsilon} + \frac{1 - \epsilon\log_2\left(\frac{1}{2}\frac{1}{\epsilon}\right) - 2\epsilon}{1-\epsilon}\log_2(2\rho) + \log_2(1-\epsilon) + 1, \tag{26}$$

which is, to first order,

$$\log_2(\Gamma) = 2 + \log_2(\rho) + \log_2(N) + (\log_2(N) - 1)\,\epsilon - \log_2\left(\frac{1}{\epsilon}\right)\log_2(2\rho)\epsilon + \text{(higher order terms)}. \tag{27}$$

Taking the derivative with respect to ϵ gives

$$\frac{\partial \log_2(\Gamma)}{\partial \epsilon} = \log_2(N) - 1 + \frac{\log_2(2\rho)}{\log(2)} - \frac{\log_2(2\rho)}{\log(2)}\log_2\left(\frac{1}{\epsilon}\right), \tag{28}$$

which we can equate to zero to find the optimal ϵ^* that minimises Γ as

$$\log_2(\epsilon^*) = -\frac{\log(2)}{\log_2(2\rho)}(\log_2(N) - 1) - 1. \tag{29}$$

The second order derivative $\frac{\partial^2 \log_2 \Gamma}{\partial \epsilon^2}$ is indeed positive, confirming that ϵ^* corresponds indeed to a minimum of Γ. Because $\epsilon = 2^{-(p+1)}$, the optimal p^* is given by

$$p^* = -\frac{\log(2)}{\log_2(2\rho)}(\log_2(N) - 1), \tag{30}$$

which scales like $\log(N)$, as alluded in the end of the previous section.

5 Estimation of the coefficient α

As explained in the main paper, and derived for a special case in the previous section, the optimal number of hierarchical levels scales logarithmically as $p^* \sim \alpha \cdot (n-1)$, where $n = \log_2 N$. Consider Figure 3 in the main paper. We see that the asymptotic regime (π is constant) starts roughly around $N = 2^5$, at the beginning of the $p^* = 4$ layer. The $p^* = 5$ layer then only occurs at $N = 2^{11}$, such that we estimate a linear slope of $\alpha \approx (5-4)/((11-1)-(5-1)) \approx 0.17$. Let us denote this estimate as $\hat{\alpha}(p^* = 4, p^* = 5)$ or just $\alpha(4, 5)$ for short. Then, we derive a more robust estimate of α by averaging across pairs $\alpha(i, j)$ for all $i \neq j$ that lie in the asymptotic (constant π) region.

6 Isolated structures

In the main paper, we have compared the output of an optimal arrangement of N individuals into a hierarchical structure against the output of N isolated individuals. But there may be yet another branch of optima, which generate an even larger output. That solution consists of some K isolated companies, that do not interact with one another. Specifically, we can write

$$\Pi_K \equiv \sum_{i=1}^{K} \Pi(N_i), \tag{31}$$

where $\sum_{i=1}^{K} N_i = N$ and each $\Pi(N_i)$ is the production that results from the optimal configuration of N_i individuals. We call the $\Pi(N_i)$ the production of the i-th (isolated) hierarchy.

A sufficient condition to check for the existence of such isolated solutions is by observing whether Π is concave or convex in N. From figure 2, we can see that Π is convex for small N, then turning concave and reaching asymptotical linearity, such that we may not draw a conclusion yet. We denote by N^* the first point at which $\Pi''(N) = 0$, i.e. where the function

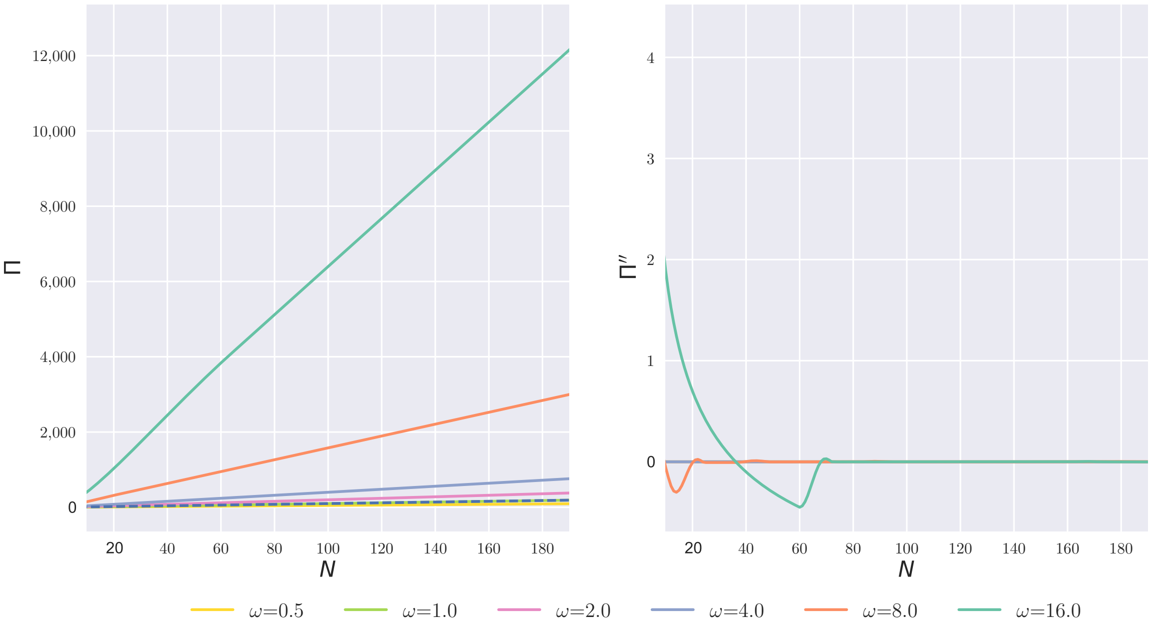

Figure 2: The left panel shows Π as a function of N for a military hierarchy (cf. section 3) with parameters $\beta = 1.5, \rho = 1$ and different ω values. The right panel shows the second-order derivative Π'' (obtained by applying a Savitsky-Golay filter). We can see from the right figure that Π is first convex, then turns concave, then briefly convex again before it asymptotically reaches linearity. (The minor last humps before reaching $\Pi'' = 0$ in the right figure are not due to a change in sign but just a numerical fluctuation induced by the filter.)

switches from convex to concave. For instance, in figure 2, this would be at roughly $N^* \approx 35$ for the $\omega = 16$ line. For $N \leqslant N^*$, isolated structures are sub-optimal, as Π is convex. We

thus consider a point $N > N^*$ and write

$$\Pi(N) = \int_0^N \mathrm{d}x\ \Pi'(x) \tag{32a}$$

$$= \int_0^{N^*} \mathrm{d}x\ \Pi'(x) + \int_{N^*}^{N} \mathrm{d}x\ \Pi'(x) \tag{32b}$$

$$= \Pi(N^*) + \int_{N^*}^{N} \mathrm{d}x\ \Pi'(x). \tag{32c}$$

We cannot increase Π by splitting below N^*. But can we increase by splitting it above N^*? The answer boils down to the question whether

$$\int_0^{N-N^*} \mathrm{d}x\ \Pi'(x) \overset{?}{<} \int_{N^*}^{N} \mathrm{d}x\ \Pi'(x). \tag{33}$$

If (33) holds true, then a unified organization is preferred over two isolated firms. We have checked numerically this condition (33) in figure 3. Interestingly, we can see that isolated economies can be beneficial, however, only at the transition to the case of overall negative output. As such, this case is of less relevance.

References

[1] E. Jones, T. Oliphant, and P. Peterson. SciPy: open source scientific tools for Python. 2014.

[2] Dieter Kraft. A software package for sequential quadratic programming. *Forschungsbericht - Deutsche Forschungs- und Versuchsanstalt fur Luft- und Raumfahrt*, 1988.

[3] J. Toulouse, G.and Bok. Principe de moindre difficulté et structures hiérarchiques. *Revue française de sociologie*, 19(3):391–406, 1978. doi: 10.1017/CBO9781107415324.004.

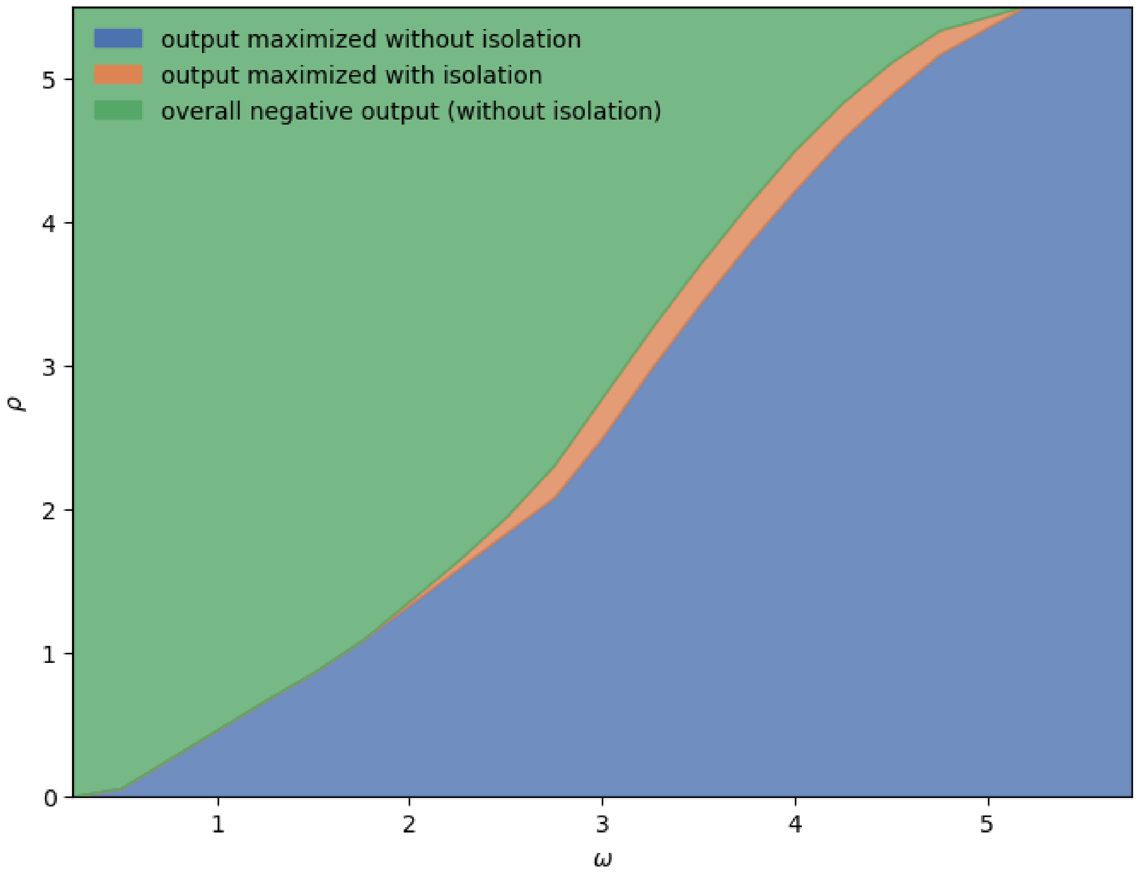

Figure 3: We have checked condition (33) for a military organization with $\beta = 1.5$ and various values of ω and ρ. Specifically, we have calculated $\Pi(N)$ from $N = 1$ up to $N = 400$. We then approximate Π' and Π'' using a Savitzgy-Golay filter (cf. figure 2). The second derivative is used to determine N^* numerically, and Π' is used to carry out the integration in (33). Three regimes are distinguished: (1) The overall output is negative (green regime), which is a somewhat degenerate case, since hierarchies are not preferred to begin with. However, isolated individual firms may still lead to a positive output, if re-structured correctly. We do not investigate this here further. (2) Structuring all N individuals into one hierarchical structure leads to greater overall output as compared to isolated substructures (blue regime). (3) Isolated substructures increase overall output, as compared to case (2) (orange regime). This case if only of relevance in the transition regime to negative overall output.
